# Pronounced Role of Lithium‐Controlling Polymer in Water‐Processable/Halogen‐Free All‐Solid‐State Electrolytes for Lithium Supercapacitors

**DOI:** 10.1002/advs.202417745

**Published:** 2025-04-17

**Authors:** Deepu Murukadas, Hwajeong Kim, Youngkyoo Kim

**Affiliations:** ^1^ Organic Nanoelectronics Laboratory and KNU Institute for Nanophotonics Applications (KINPA) Department of Chemical Engineering, School of Chemical Engineering and Applied Chemistry Kyungpook National University Daegu 41566 Republic of Korea; ^2^ Department of Energy Convergence & Climate Change and Institute for Global Climate Change and Energy Kyungpook National University Daegu 41566 Republic of Korea; ^3^ Priority Research Center Research Institute of Environmental Science & Technology Kyungpook National University Daegu 41566 Republic of Korea

**Keywords:** all‐solid‐state electrolytes, bPEI, lithium supercapacitors, PSSA, water‐processable

## Abstract

Polymeric solid‐state electrolytes (SSEs) with environmentally friendly processes deliver safer and cleaner energy storage devices without fires and leakages than conventional liquid electrolytes. Here, water‐processable halogen‐free polymeric SSEs are demonstrated with high ion conductivity (≈6 mS cm^−1^), prepared from aqueous solutions consisting of branched poly(ethylene imine) (bPEI), lithium hydroxide (LiOH), and poly(4‐styrene sulfonic acid) (PSSA). The bPEI:LiOH:PSSA (PLP) SSEs with various PSSA molar ratios are applied to asymmetric supercapacitors with graphite‐based anodes and indium tin oxide (ITO) counter electrodes. The PSSA molar ratio strongly affected the ion conductivity of PLP SSEs, leading to a maximum at PSSA = 40 mol%, owing to the role of PSSA in controlling the size of LiOH domains for better Li^+^ transport pathways. The enhanced ion conductivity enabled PLP‐supercapacitors to build a high potential of 2.24 V at PSSA = 40 mol%, compared to 1.64 V at 0 mol%, upon galvanostatic charge/discharge at a current density of 0.2 mA g^−1^. The endurance test shows that the supercapacitors with the PLP SSEs (PSSA = 40 mol%) can function stably with high capacitance retention (96.2%) for more than 5000 cycles, and ≈80% capacitance retention at 80 °C, supporting their practical use in high‐safety supercapacitors and batteries.

## Graphical abstract

Water‐processable solid‐state electrolytes, composed of branched‐poly(ethylene imine), lithium hydroxide, and poly(4‐styrene sulfonic acid), exhibit an ion conductivity of ≈6.0 mS cm^−1^ and enable high‐performance lithium supercapacitors with excellent endurance (ca. 96% at 25 °C and 80% at 80 °C) upon 5000 charging/discharging cycles.

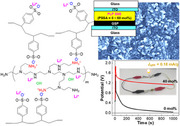

## 1 Introduction

Polymeric solid‐state electrolytes (SSEs), featuring mechanical flexibility and coating processability, have attracted keen interest in next‐generation energy storage devices including supercapacitors and rechargeable batteries.^[^ ^1^, ^2^, ^3^, ^4^, ^5^, ^6^ ^]^ Compared to conventional liquid‐state electrolytes (LSEs), polymeric SSEs have the advantage of design freedom for device shapes due to high stability and safety without leakages and fires which are serious issues for LSEs.^[^ ^7^, ^8^, ^9^, ^10^, ^11^ ^]^ However, the major drawback of polymeric SSEs is low ion conductivity because ions such as lithium cations (Li^+^) are vulnerable to facing physical resistances caused by polymeric chains that are almost fixed in the solid‐state frames.^[^ ^12^, ^13^, ^14^, ^15^, ^16^, ^17^ ^]^

To improve the ion conductivity of polymeric SSEs, various approaches have been attempted (see Table S1, Supporting Information): 1) adding inorganic/ceramic materials, 2) ionic liquids, 3) plasticizers, and 4) incorporating secondary polymer materials.^[^ ^18^, ^19^, ^20^, ^21^, ^22^, ^23^, ^24^, ^25^, ^26^ ^]^ In the case of adding inorganic materials such as zeolites, lithium lanthanum zirconium oxide (LLZO), and lithium aluminum titanium phosphate (Li_1+x_Al_x_Ti_2−x_(PO_4_)_3_), the resulting hybrid composite‐type SSEs could have an improved ion conductivity of 10⁻⁴–10⁻^3^ S cm^−1^.^[^ ^18^, ^19^ ^]^ An ionic liquid, 1‐butyl‐3‐methylimidazolium bis(trifluoromethylsulfonyl)imide (BMIMTFSI), was used to develop a solid polymer electrolyte consisting of poly(ethylene oxide) (PEO) and lithium bis(trifluoromethanesulfonyl)imide (LiTFSI), achieving an ion conductivity of 1.5×10⁻⁴ S cm^−1^ at 30 °C.^[^ ^20^ ^]^ The addition of succinonitrile (SN), a plasticizer, to PEO and a 3D glass fiber (GF) framework, led to an ion conductivity of 2.85×10⁻⁴ S cm^−1^.^[^ ^21^ ^]^ A polymer blend electrolyte, which contains PEO, poly(vinylidene fluoride‐co‐hexafluoropropylene) (PVdF‐HFP), and ammonium bromide (NH₄Br), delivered a maximum ion conductivity of 2.55×10⁻⁴ S cm^−1^ at 303 K.^[^ ^22^ ^]^

With increasing interest in environmentally friendly processes for energy storage devices, water‐processable polymeric SSEs have been spotlighted, but only a few works reported on the use of fully non‐hazardous materials (see Table S1, Supporting Information). Halogen‐containing lithium salts, such as lithium chloride (LiCl) and lithium perchlorate (LiClO_4_) for water‐processable electrolytes, are classified as hazardous under the Treaty on European Union (TFEU).^[^ ^27^, ^28^ ^]^ On this account, our recent works showed that branched poly(ethylene imine) (bPEI) and lithium hydroxide (LiOH), well soluble in water and classified as environmentally non‐hazardous, could deliver polymeric SSEs with reasonable ion conductivity.^[^ ^29^ ^]^ However, less attention has been paid to combining two different water‐soluble polymers that play individual roles in polymeric SSEs. Interestingly, poly(styrene sulfonic acid) (PSSA), a well‐known water‐soluble and environmentally non‐hazardous polymer, has not been employed for such polymer blend‐type SSEs even though it was applied for various applications such as gas sensors, ion‐exchange membranes, electrochemical capacitors' cathodes, and fuel cells.^[^ ^30^, ^31^, ^32^, ^33^, ^34^, ^35^, ^36^, ^37^ ^]^

In this work, we demonstrate water‐processable polymeric SSEs with an ion conductivity of ≈6 mS cm^−1^ and their applications to supercapacitors with the asymmetric configuration of electrodes. The polymeric SSEs were prepared by adding poly(styrene sulfonic acid) (PSSA) in the bPEI:LiOH binary electrolyte mixtures in aqueous solutions. The molar ratio of PSSA to bPEI (based on repeating units) varied up to 60 mol%. The resulting bPEI:LiOH:PSSA (PLP) SSEs were sandwiched between graphite‐based anodes and indium‐tin‐oxide (ITO) cathodes, leading to asymmetric supercapacitors. The present PLP SSEs can be mass‐produced using water without environmental hazardousness and are considered not to be seriously harmful to human skin because of neutralized states between acidic (PSSA) and basic (bPEI and LiOH) components.

## 2 Results and Discussion

**Figure 1:**
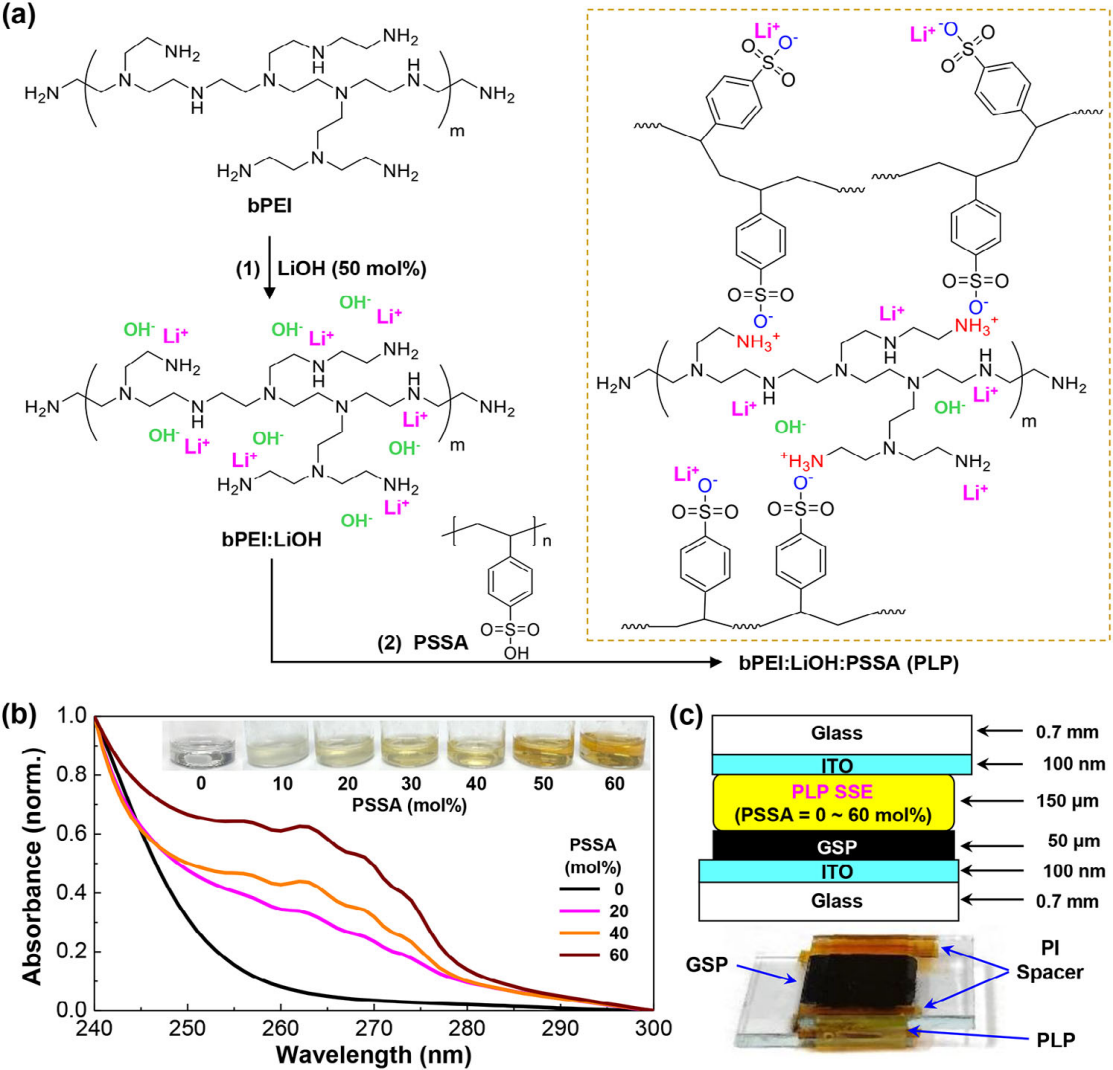
a) Scheme to prepare bPEI:LiOH:PSSA (PLP) electrolytes using water as a solvent: (1) LiOH addition to the aqueous bPEI solution, (2) PSSA addition to the bPEI:LiOH mixture solution. Note that the positions of Li^+^ and OH^−^ were arbitrarily displayed. b) Representative optical absorption spectra for the PLP SSE films according to the PSSA molar ratio (inset: photographs of PLP solutions). c) Device structure of asymmetric supercapacitors with the PLP SSEs (PSSA = 0–60 mol%) (bottom: photograph of the PLP supercapacitor fabricated by sandwiching both PLP‐coated GSP/ITO‐glass (top) and PLP‐coated ITO‐glass (bottom)).

As shown in **Figure** **1**a, aqueous solutions of binary electrolytes (bPEI:LiOH) were first prepared by mixing LiOH and bPEI at the fixed molar ratio of LiOH (50 mol% based on the repeating unit of bPEI polymer).^[^ ^29^ ^]^ To the bPEI:LiOH solutions, the PSSA solution was added by varying the molar ratio of PSSA (0–60 mol% based on the repeating unit of bPEI), which led to the bPEI:LiOH:PSSA (PLP) ternary solutions. As the PSSA ratio increased, the color of PLP solutions gradually changed to a deep yellow (see photograph in Figure 1b), as supported by the increased optical absorption of PLP films in the wavelength (λ) range of 250–280 nm (Figure 1b; Figure S1a, Supporting Information for all spectra). These optical absorptions at λ = 250–280 nm can be attributed to the PSSA polymer that absorbs photons in this wavelength range (see Figure S1b, Supporting Information). The PLP solutions were drop‐cast onto the Graphite‐ Super P‐Li‐ PVdF (GSP)‐coated ITO‐glass and bare ITO‐glass substrates, which were dried and sandwiched, leading to asymmetric supercapacitors as displayed in Figure 1c (see details in the experimental section).

**Figure 2:**
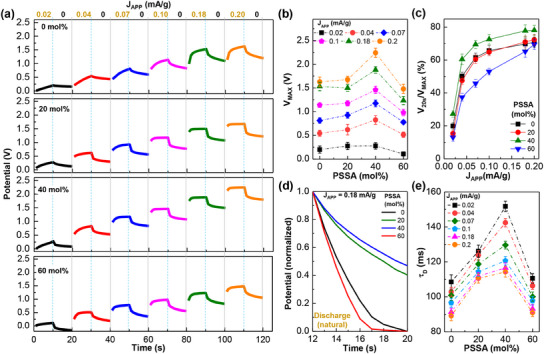
a) Galvanostatic charging/discharging (GCD) curves for the supercapacitors with the PLP SSEs upon the consecutive stepwise increase of applied current density (J_APP_) at the same charging/discharging duration time (10 s). b) The maximum potential (V_MAX_) as a function of PSSA molar ratio (0–60 mol%) for the PLP‐supercapacitors according to J_APP_ (0.02–0.2 mA g^−1^). c) The retention of potential after discharging for 10 s (20 s from the beginning of charging operation) (defined as V_20s_/V_MAX_) as a function of J_APP_ according to the PSSA molar ratio. d) Comparison of normalized discharging curves according to the PSSA molar ratio (J_APP_ = 0.18 mA g^−1^). e) Decay time constant (**τ_D_**) as a function of PSSA molar ratio (see the normalization process in the experimental section of Supporting Information).

The performance of devices with the PLP electrolytes was examined by employing galvanostatic charging and discharging (GCD) measurements. As shown in **Figure** **2**a, the first (initial) charging operation for 10 s at the current density (J_APP_) of 0.02 mA g^−1^ delivered an increased potential for all devices, even though the potential level (0.2–2.24 V) depended on the PSSA molar ratio. The increased potential (voltage) of devices was slowly reduced during the natural discharging operation (DCO) without any current bias (J_APP_ = 0 mA g^−1^) (note that the potential (voltage) drop at the beginning of discharge operations can be attributed to electrostatic discharge (ESD) effects).^[^ ^38^ ^]^ The consecutive charging operations with the stepwise increase in current densities (up to 0.2 mA g^−1^) resulted in gradually enhanced potentials. This result indicates that the present devices with the PLP SSE layers act to store electrical energy like capacitors.

The detailed analysis showed that the maximum potential (V_MAX_) was achieved at PSSA = 40 mol% irrespective of applied current density (note that V_MAX_ = 2.24 V at J_APP_ = 0.2 mA g^−1^) (see Figure 2b). This maximum potential (2.24 V) can be assigned to the highest value among non‐halogen‐type polymer‐based electrolytes reported to date (Table S1, Supporting Information). In more detail, the maximum potential was marginally higher at PSSA = 20 mol% than 0 mol%, whereas it became even lower at PSSA = 60 mol% than 0 mol% (no PSSA). To understand the potential retention after charging for 10 s, the ratio of the potential at 20 s (10 s after discharging) to the maximum potential (V_20s_/V_MAX_) was plotted as a function of J_APP_ in Figure 2c. This analysis uncovered the highest potential retention at PSSA = 40 mol% over the whole J_APP_ range but the lowest one at PSSA = 60 mol%, which is supported by the slowest decay in potential at PSSA = 40 mol% in the discharging stage (see Figure 2d). Further in‐depth analysis, employing an exponential decay equation, revealed that the highest decay time constant (τ_D_), indicating the slowest decay, was achieved at PSSA = 40 mol% irrespective of J_APP_ (see Figure 2e including the decay equation). Here, the faster potential decay (the lower decay time constant) at higher J_APP_ might reflect the relatively higher number of lithium cations unstably captured in the GSP anodes. As a result, the highest energy density of ca. 2.33 mWh g^−1^ could be achieved for the devices with the PLP SSEs (PSSA = 40 mol%) (see Figure S2, Supporting Information).

**Figure 3:**
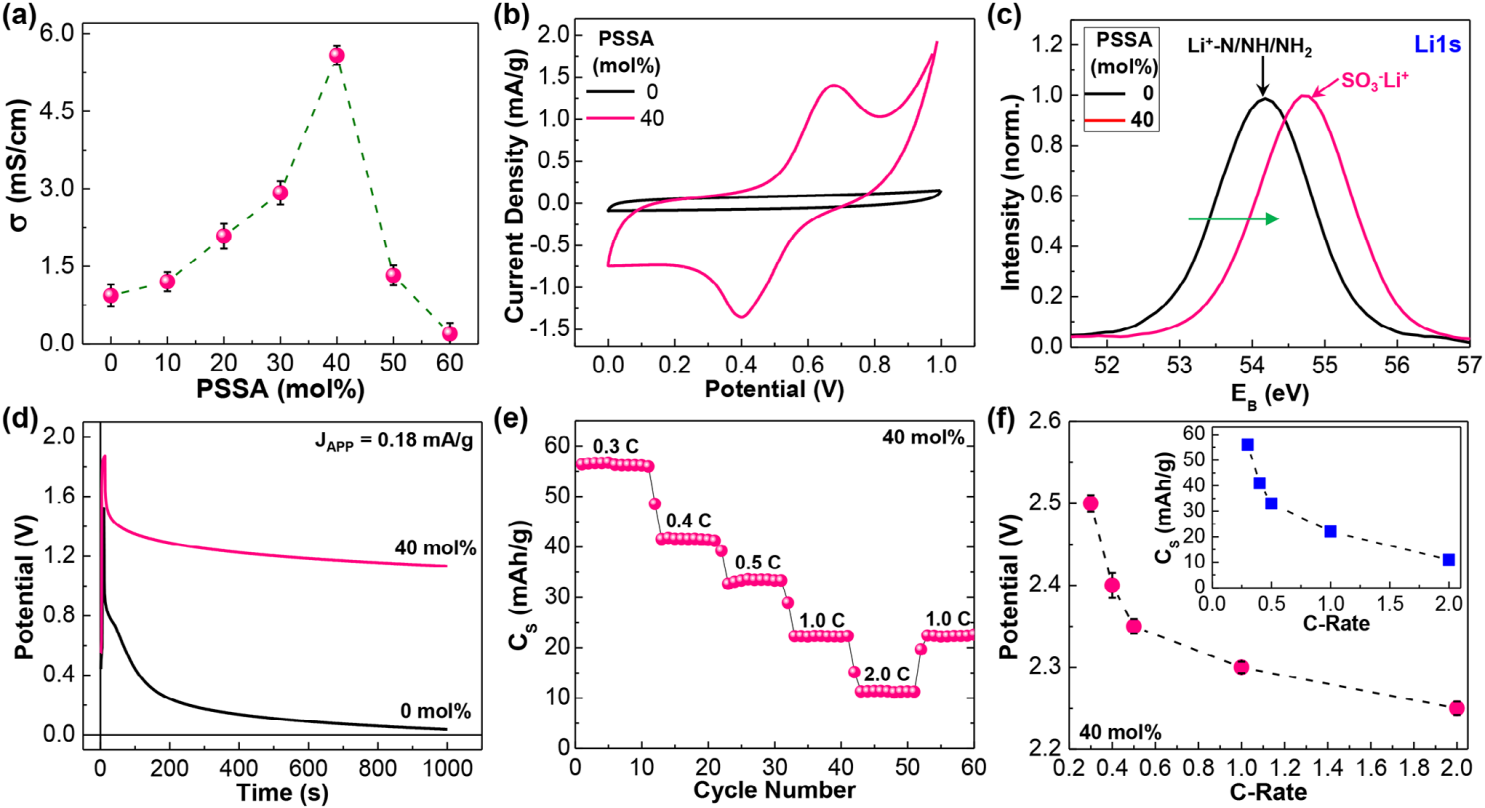
a) Ionic conductivity (σ) as a function of PSSA molar ratio (0–60 mol%) for the PLP SSEs in the present device structure. b) Representative cyclic voltammetry (CV) curves (SR = 1.0 V s^−1^) for the supercapacitors with the PLP SSEs according to PSSA molar ratio (0 and 40 mol%) (see other CV curves in Figure S6a, Supporting Information). c) Li1s XPS spectra (normalized) of the PLP SSE films (PSSA = 0 and 40 mol%) (the green arrow indicates the direction of peak shift). d) Comparison of long‐term discharge characteristics for the supercapacitors with the PLP SSEs (0 and 40 mol%) (discharging at J_APP_ = 0 mA g^−1^ after charging for 10 s at J_APP_ = 0.18 mA g^−1^). e) Specific capacity (C_S_) as a function of C‐rate (0.3, 0.4, 0.5, 1, and 2 C) for the PLP40‐SCs (note that 10 charging/discharging cycles for varied periods were conducted at each C‐rate). f) Device potential (40 mol%) as a function of C‐rate (inset: specific capacity as a function of C‐rate).

This significant change in GCD performances according to the PSSA molar ratio can be attributed to the ion conductivity of PLP SSEs, which showed the highest value (≈5.6 mS cm^−1^) at PSSA = 40 mol% and the lowest value (0.2 mS cm^−1^) at 60 mol% (see **Figure** **3**a; Figure S3a, Supporting Information). In other words, the highest maximum potential (V_MAX_) could be made at PSSA = 40 mol% due to the fastest transport of lithium cations to the GSP layers. Note that the present ion conductivity of 5.58 mS cm^−1^ at PSSA = 40 mol% is one of the highest values for polymeric SSEs reported to date (see Table S1, Supporting Information).^[^ ^39^ ^]^ Note that the ion conductivity of the PLP SSEs (PSSA = 40 mol%) changed to 3.45 mS cm^−1^ at 80 °C (see Figure S3b, Supporting Information), which will be discussed again for the stability analysis below. To assess any residual water, the PLP SSE films were carefully detached from the devices (supercapacitors) and brought into contact with tissue paper. As illustrated in Figure S4 (Supporting Information) (see video clip), no wetted state was observed on the tissue paper, suggesting that the surface of PLP films had almost completely dried. Additionally, the PLP films were further dried at 140 °C for 40 mins to investigate weight loss by water extraction (see Figure S5, Supporting Information). The result showed that the weight change was only 0.06 wt%, supporting that an extremely small amount of water might be included in the PLP films (see Figure S5, Supporting Information). As shown in Figure 3b, the superior GCD performance for the devices with the PLP SSEs (PSSA = 40 mol%) is also supported by the much wider current density window of cyclic voltammetry (CV) curves at PSSA = 40 mol% than 0 mol%, indicative of more efficient lithium cation transport at PSSA = 40 mol%. Note that the redox peaks at ca. 0.40 V and 0.68 V can be assigned to the reduction and oxidation of the SO_3_^−^Li^+^ units in the PLP SSEs (see all CV curves in Figure S6a, Supporting Information). The formation of the SO_3_^−^Li^+^ units was confirmed from the XPS spectra that exhibited a clear Li1s peak at 54.7 eV corresponding to the SO_3_^−^Li^+^ groups (see Figure 3c; other XPS peaks in Figure S7, Supporting Information).

Interestingly, the current density trend for the redox peaks (CV curves) was well aligned with the ion conductivity trend (Figure S6b, Supporting Information), indicating the critical influence of PSSA contents on the GCD performances of devices, which will be discussed further in the mechanism section. In addition, as displayed in Figure 3d, the potential during self (natural) discharging operations (after charging) decayed very slowly at PSSA = 40 mol% compared to 0 mol%. This result implies that the lithium cations might be more efficiently bound (captured) to the GSP anodes at PSSA = 40 mol% than 0 mol% due to the effective role of PSSA components including the fast lithium cation transport. The charging and discharging characteristics of the devices with PLP SSEs (PSSA = 40 mol%), abbreviated as PLP40‐SCs, were examined by varying the applied current rate (C‐rate) between 0.3 C and 2.0 C. As shown in Figure 3e, the specific capacity (Cs) of devices was well‐maintained at the same C‐rate upon the consecutive 10 charging/discharging cycles for the whole C‐rate range. The maximum specific capacity was measured to be 56.4 mAh g^−1^ at the C‐rate of 0.3 C, comparable to the typical performance of graphite‐based lithium supercapacitors.^[^ ^40^ ^]^ As the C‐rate increased from 0.3 to 2.0 C, the specific capacity gradually decreased from 56.4 mAh g^−1^ (0.3 C) to 11.3 mAh g^−1^ (2.0 C). This trend can be attributed to the limited lithium cation transport in the PLP SSEs (PSSA = 40 mol%), which cannot meet the high rate of current flows applied to the devices, as generally observed from lithium‐ion batteries.^[^ ^41^ ^]^ However, the specific capacity was restored to 22.3 mAh g^−1^ from 11.3 mAh g^−1^ upon decreasing the high C‐rate from 2.0 to 1.0 C, supporting device stability under various charging/discharging conditions. As plotted in Figure 3f, the potential of devices was measured as 2.5 V at 0.3 C but decreased gradually as the C‐rate increased. Here, it is noted that the device potential decreased by only 10% (from 2.5 V at 0.3 C to 2.25 V at 2.0 C) even though the C‐rate increased by ca. 660% (from 0.3 to 2.0 C) (see Figure 3f inset for the specific capacity change as a function of C‐rate).

**Figure 4:**
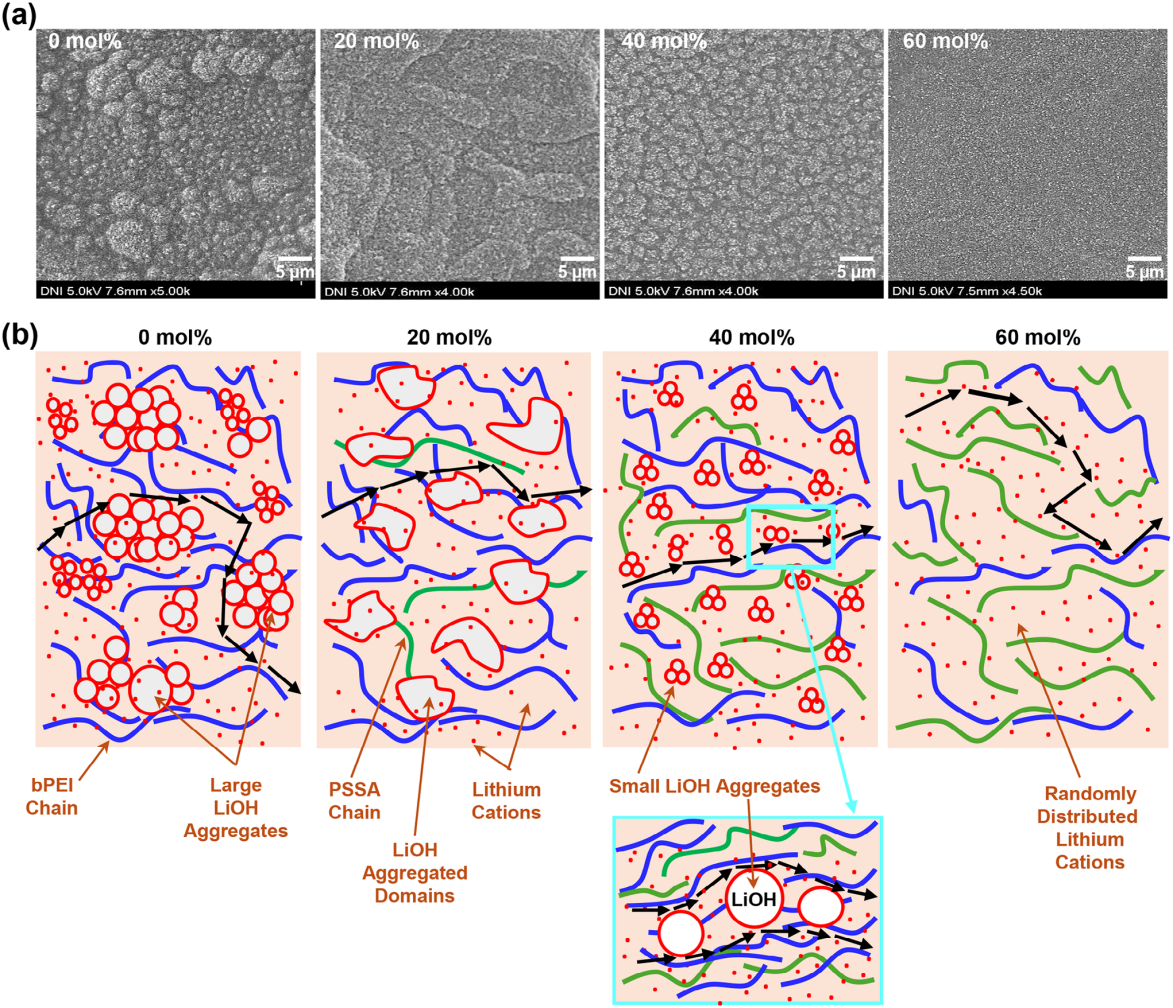
a) FE‐SEM images for the PLP SSE films (PSSA = 0, 20, 40 and 60 mol%). b) Morphological illustration for the transport mechanism of lithium cations in the PLP SSEs according to the PSSA molar ratio (note that the arrows in black denote the transport pathway of lithium cations).

To understand the mechanism of lithium cation transport in the PLP SSEs, the surface morphology of PLP films was examined with field emission–scanning electron microscopy (FE‐SEM). As shown in **Figure** **4**a, the bPEI:LiOH film (PSSA = 0 mol%) delivered several micro‐domains with various sizes between <1 µm and ca. 7 µm, which might be LiOH aggregates caused by phase separations between bPEI chains and excess LiOH molecules [left except LiOH (i.e., Li^+^) bound to the nitrogen atoms of bPEI chains].^[^ ^42^ ^]^ Adding PSSA (20 mol%) changed the morphology to have a stretched domain in the presence of finer aggregates (ca. 7 µm) distributed randomly. At PSSA = 40 mol%, irregularly shaped micro‐domains (ca. 2–5 µm size) with several nanoparticles were formed over the whole surface of the PLP films (see Figure S8, Supporting Information). Interestingly, at the highest PSSA content (60 mol%), the entire surface of PLP films became much finer in the presence of randomly distributed nanoparticles (ca. 200 nm).

This morphology change is further explained by the analysis of Raman spectra, which can provide information on molecular‐level interactions among the three components in the PLP films (see Figure S9, Supporting Information). In detail, as shown in Figure S9a (Supporting Information), the C─H stretching region (2800–3000 cm⁻¹) exhibits two distinct peaks, corresponding to the symmetric (≈2840–2880 cm⁻¹) and asymmetric (≈2920–2970 cm⁻¹) C─H stretching modes. These vibrations are considered to originate from the aliphatic chains of bPEI (see the peaks at PSSA = 0 mol%). As the PSSA content increased, the two peaks almost gradually shifted toward a low wavenumber direction, indicating alterations in the molecular‐level environment of bPEI chains (note that the asymmetric stretching peak at PSSA = 20 mol% was moved oppositely). This result supports that the bPEI chains were influenced by the addition of PSSA polymers. Interestingly, as the PSSA content increased, the N─H stretching peak (3200–3500 cm⁻¹) also gradually shifted toward a low wavenumber direction, which can be merely attributed to co‐effects of C─H stretching change but might inform the environmental change of lithium cation‐nitrogen (Li^+^‐N) interactions in the bPEI domains by the added PSSA polymer (Figure S9b, Supporting Information). In other words, the PSSA polymer chains might affect the interaction between lithium cations and bPEI chains (see the details below for the proposed mechanism in Figure 4b). Note that the gradually increasing intensity of C═C stretching (≈1550–1650 cm⁻¹) evidences the presence of PSSA chains in the PLP films (Figure S9c, Supporting Information).

This morphology investigation finds a strong effect of PSSA content on the morphology of PLP films, which can be closely related to ion conductivity and GCD performance. Based on this morphology change, a mechanism for lithium cation transport in the PLP SSEs can be proposed as illustrated in Figure 4b: 1) At PSSA = 0 mol%, owing to the interfering role of large LiOH micro‐domains, a limited number of lithium cations can transport by hopping through the nitrogen atoms in the bPEI chains (no PSSA role); 2) At PSSA = 20 mol%, the ion conductivity slightly increases because of the lessened physical impedance due to the restructured morphology (stretched domains but reduced aggregate size) via small scale acid‐base reactions between PSSA and LiOH leading to the SO_3_^−^Li^+^ units; 3) At PSSA = 40 mol%, by sufficient acid‐base reactions between PSSA and LiOH, the ion conductivity greatly increases due to the well‐organized morphology with finer LiOH aggregates of which polar surfaces (OH groups mainly) might help the hopping transport of lithium cations of which numbers might be made by a balance between N‐Li^+^ units in bPEI parts and SO_3_^−^Li^+^ units in PSSA parts according to the CV results); 4) At PSSA = 60 mol%, the ion conductivity largely decreases owing to the reduced number of mobile lithium cations in bPEI parts because the relatively large amount of PSSA chains bind the lithium cations by acid‐base reactions with LiOH in solutions.

**Figure 5:**
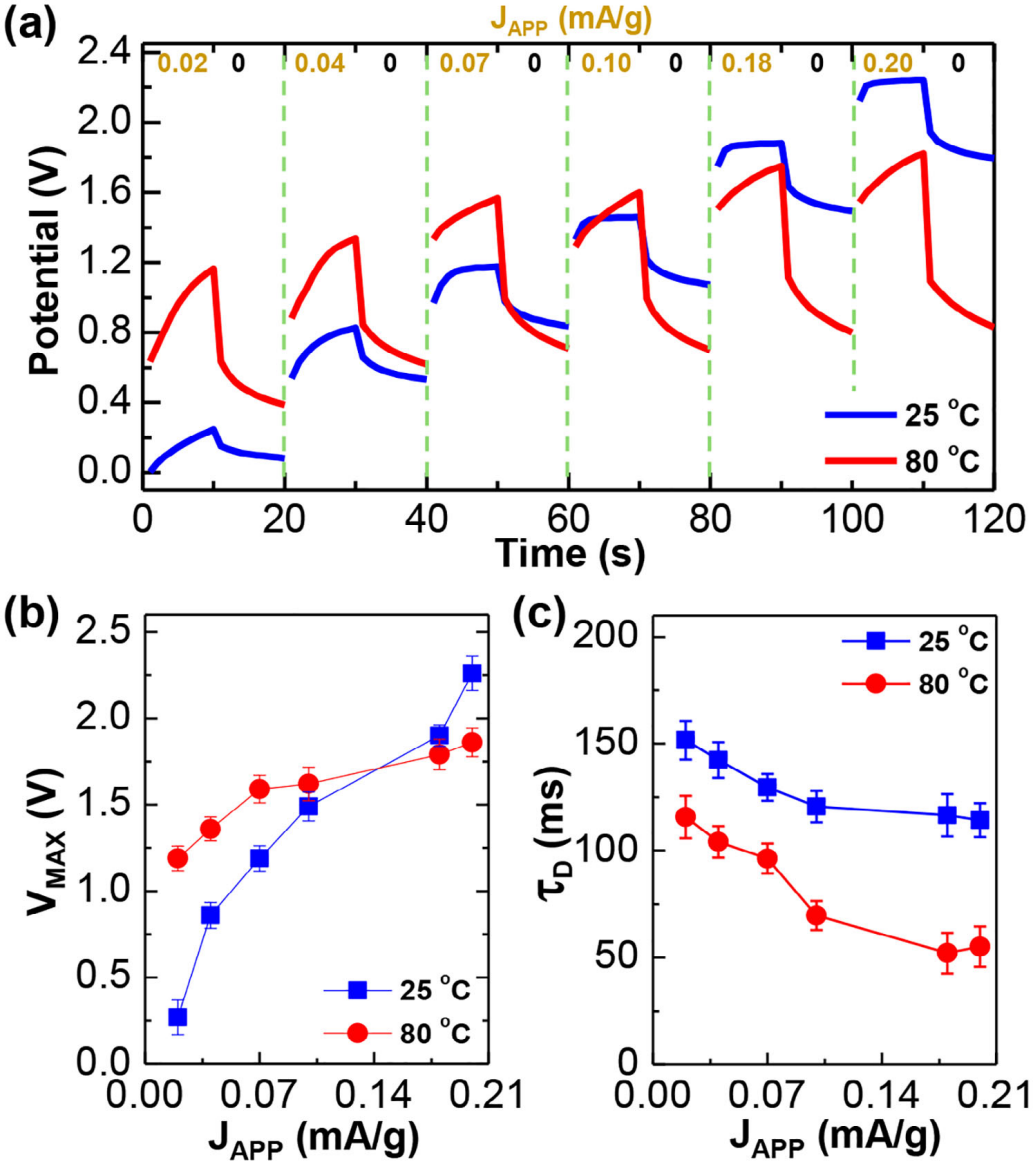
a) GCD curves of PLP40‐SCs operated at 25 °C and 80 °C upon charging/discharging cycles with a stepwise increase of J_APP_. b) Maximum potential (V_MAX_) as a function of J_APP_ at 25 °C and 80 °C. c) Decay time constant (τ_D_) as a function of J_APP_ at 25 °C and 80 °C. Note that the data in (b) and (c) were extracted from the GCD curves in (a). The decay time constant was obtained using V(t) = V_0_​ + $e^{-t/\tau_{D}}$, where V(t) and V_0_ denote potential at a time (t) and t = 0 s (initial point of discharging), respectively.

To further assess the stability of PLP40‐SCs, the elevated temperature condition was applied for the GCD test with gradually increasing applied current densities. As shown in **Figure** **5**a, the device potential during charging at J_APP_ = 0.02 mA g^−1^ was higher at 80 °C than at 25 °C. This trend was maintained up to J_APP_ = 0.1 mA g^−1^ and reversed at the higher J_APP_ (0.18 and 0.2 mA g^−1^). The high potential built initially at 80 °C (J_APP_ = 0.02–0.1 mA g^−1^) can be attributed to the promoted transport of lithium cations by increased relaxations (vibrations) of polymer chains.^[^ ^43^ ^]^ However, as the GCD operations consecutively proceeded up to J_APP_ = 0.2 mA g^−1^ at 80 °C, the potential increase might be limited owing to several reasons including the change of anode‐PLP interfaces leading to the reduced ratio of charged states (V_20s_/V_MAX_) after discharging for 10 s (see Figure 5b; Figure S10a, Supporting Information) and the potential drop by electrostatic discharge (V_ESD_) (see Figure S10b, Supporting Information). As plotted in Figure 5c, the decay time constant (τ_D_) for the discharging part was relatively lower at 80 °C than at 25 °C, which may reflect a faster extraction and transport of lithium cations in the PLP SSEs at a high temperature, as frequently observed for various electrolytes.^[^ ^44^ ^]^

**Figure 6:**
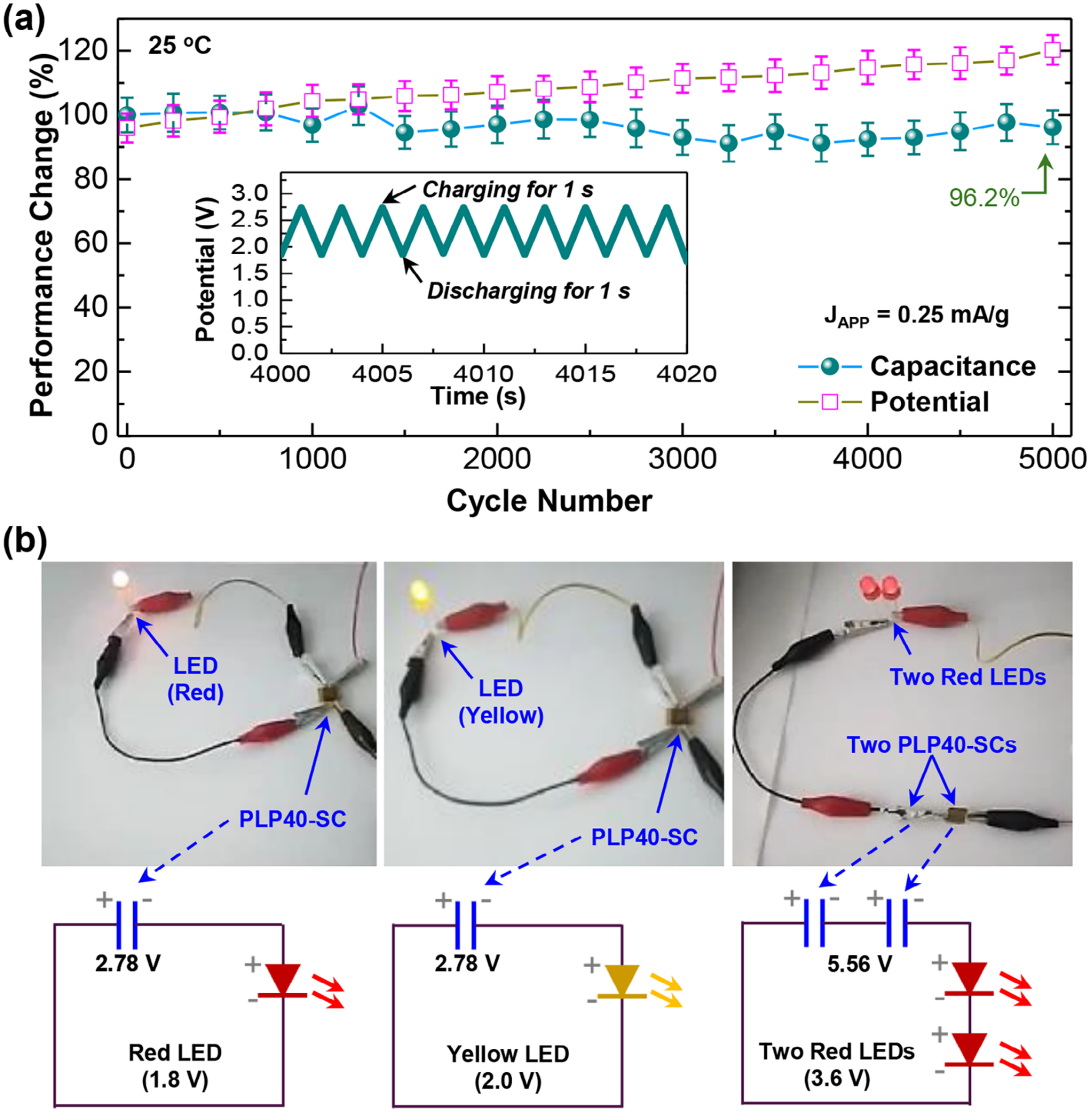
a) Change of capacitance and potential as a function of cycle number (charging for 10 s at J_APP_ = 0.25 mA g^−1^ and discharging for 10 s at J_APP_ = 0 mA g^−1^ at 25 °C) (see inset for the part of long‐term raw data and full data at 25 °C and 80 °C in Figure S11, Supporting Information). b) Demonstration of LED operations using the present lithium supercapacitors (PLP40‐SCs): Note that the marked voltages of PLP40‐SCs in the equivalent diagrams correspond to the initial (as‐charged) potentials (see video clips attached as Figure 6a_video.mp4, Figure 6b_video.mp4, and Figure 6c_video.mp4, Supporting Information).

Based on the GCD and analysis results, the PLP40‐SCs were subjected to endurance tests by applying 5000 charging/discharging cycle operations at J_APP_ = 0.25 mA g^−1^ at 25 °C. As shown in **Figure** **6**a, the potential of PLP40‐SCs was changed slightly, by ca. 12% after 5000 cycles (see Figure S11a, Supporting Information for the potential change with time). However, interestingly, the capacitance of PLP40‐SCs was well retained by 96.2% after 5000 cycles. This small change (only a 3.8% reduction) in capacitance even after 5000 charging/discharging cycles supports the stable transport of lithium cations in the PLP SSEs (PSSA = 40 mol%), leading to long‐term endurance in devices, which is one of the excellent stability results reported on the graphite‐based supercapacitors.^[^ ^45^, ^46^, ^47^ ^]^ At 80 °C, the initial potential of PLP40‐SCs was ca. 1.8 V, relatively lower than ca. 2.5 V at 25 °C, which can be attributed to the decreased ion conductivity at this elevated temperature (see Figure S11b, Supporting Information). Upon continuous cycling up to 5000 cycles, the potential gap between charging and discharging steps became larger at 80 °C compared to 25 °C. The maximum potential changed by ≈65% from 1.81 V (cycle = 0) to 2.98 V (cycle = 1000). This relatively large change in potential at 80 °C may reflect the limited stability at this elevated temperature. The reason can be explained by the increased vibrations of polymer chains at high temperatures, affecting the transport of lithium cations.

Finally, the present PLP40‐SCs were examined as a power source for light‐emitting diodes (LEDs). As demonstrated in Figure 6b (see video clips in Figure S12, Supporting Information), the single PLP40‐SC device was charged successfully to have 2.78 V (J_APP_ = 0.25 mA g^−1^ for 500 s) and could operate red and yellow LEDs to lit brightly. In addition, two PLP40‐SC devices were connected in series and charged to achieve the initial output voltage of 5.56 V (J_APP_ = 0.25 mA g^−1^ for 500 s), which could successfully drive two series‐connected red LEDs (see Figure 6b bottom part for the simplified equivalent circuit diagrams including typical LED operation voltages). Note that the brightness of the two series‐connected LEDs decreased with time because the output voltage from the two PLP40‐SCs dropped gradually from 5.56 to 3.8 V after 500 s (see Figure S12c, Supporting Information). This LED operation test suggests the great potential of PLP40‐SCs for practical applications.

## 3 Conclusion

In summary, water‐processable polymeric SSEs (bPEI:LiOH:PSSA ‐ PLP) were successfully prepared with various PSSA molar ratios and applied to asymmetric supercapacitors. The GCD tests revealed that the highest potential (2.24 V at J_APP_ = 0.2 mA g^−1^) and potential retention (V_20s_/V_MAX_) characteristics were achieved for the supercapacitors with PLP SSEs (PSSA = 40 mol%) (i.e., PLP40‐SCs). The C‐rate‐dependent test confirmed that the PLP40‐SCs could undergo charging/discharging operations at various current‐rate conditions. The outstanding charging/discharging performance at PSSA = 40 mol% is supported by the highest ion conductivity (5.6 mS cm^−1^) and the largest hysteresis (CV) window at this molar ratio. Such a strong influence of the PSSA molar ratio was explained by the mechanism of lithium cation transport, affected by the morphology (LiOH nano/micro‐domains) of PLP films, which is controlled by the acid‐base reactions of PSSA with LiOH. The thermal stability test uncovered that the supercapacitors with the PLP SSEs (PSSA = 40 mol%) could function normally at 80 °C despite a marginal reduction in potential retention. The endurance test showed that the capacitance of the supercapacitors (PLP40‐SCs) maintained ca. 96.2% even after 5000 charging/discharging cycles in the presence of a slight increase in device potential. The single PLP40‐SC could operate both red and yellow LEDs, while two PLP40‐SCs in series could drive two red LEDs. Conclusively, the present PLP SSEs with a lithium‐controlling polymer concept are expected to greatly contribute to further improved performance of polymeric SSEs for various energy storage devices.

## 4 Experimental Section

Experimental Details are provided in the Supporting Information

## Conflict of Interest

The authors declare no conflict of interest.

## Supporting information



Supporting Information

Supplemental Fig_S4_video

Supplemental Fig_6a_video

Supplemental Fig_6b_video

Supplemental Fig_6c_video

Supplemental Fig_S12a_video

Supplemental Fig_S12b_video

Supplemental Fig_S12c_video

## Data Availability

The data that support the findings of this study are available in the supplementary material of this article.
