# Supplementary material for: Pronounced Role of Lithium‐Controlling Polymer in Water‐Processable/Halogen‐Free All‐Solid‐State Electrolytes for Lithium Supercapacitors
Source: Adv Sci (Weinh). 2025 Apr 17;12(23):2417745. doi: 10.1002/advs.202417745 (PMC12199392; doi:10.1002/advs.202417745)
Supplement: Supplementary file 1 — Supporting Information [file ADVS-12-2417745-s005.pdf]

ADVANCED SCIENCE
Open Access

Supporting Information

for *Adv. Sci.*, DOI 10.1002/advs.202417745

Pronounced Role of Lithium-Controlling Polymer in Water-Processable/Halogen-Free All-Solid-State Electrolytes for Lithium Supercapacitors

*Deepu Murukadas, Hwajeong Kim and Youngkyoo Kim**

Supporting Information

Pronounced Role of Lithium-Controlling Polymer in Water-Processable/Halogen-Free All-Solid-State Electrolytes for Lithium Supercapacitors

Deepu Murukadas[1,2], Hwajeong Kim[1,3], Youngkyoo Kim[1,2,*]

[1]*Organic Nanoelectronics Laboratory and KNU Institute for Nanophotonics Applications (KINPA), Department of Chemical Engineering, Kyungpook National University, Daegu 41566, Republic of Korea*

[2]*Department of Energy Convergence & Climate Change, Kyungpook National University, Daegu 41566, Republic of Korea*

[3]*Priority Research Center, Research Institute of Environmental Science & Technology, Kyungpook National University, Daegu 41566, Republic of Korea*

[*]Corresponding author: Prof. Youngkyoo Kim

Email) ykimm@knu.ac.kr, Tel) +82-(0)53-950-5616

Experimental section

Materials, solutions, and pastes: Poly(styrene sulfonic acid) (PSSA, weight-average molecular weight = 75 kDa), lithium hydroxide (LiOH), The branched poly(ethylene imine) (bPEI) aqueous solution (≤1 wt%) (weight-average molecular weight = ~25 kDa, number-average molecular weight = ~10 kDa, polydispersity index (PDI) = ~2.5), poly(vinylidene fluoride) (PVdF, weight-average molecular weight = ~534 kDa), and N-methyl-2-pyrrolidone (NMP) were purchased from Sigma-Aldrich (USA) and used as received without further purification. Super P-Li (density = 1.60 g/cm^3, BET surface area = 62.0 m^2/g) and ultra-fine artificial graphite powder (average particle diameter = 30 μm) were supplied from TIMCAL (Switzerland) and SHOWA DENKO (Japan), respectively. The binary solutions of bPEI and LiOH were prepared by dissolving each in deionized (DI) water, followed by adding PSSA (PSSA molar ratio = 0, 10, 20, 30, 40, 50, and 60 mol% to the repeating unit of bPEI). The exact amount (weight ratio) of components in the PLP solutions (DI water = 1 mL) is given by bPEI:LiOH:PSSA = 0.533g:0.04g:0g (0 mol%), 0.533g:0.04g:0.05g (10 mol%), 0.533g:0.04g:0.1g (20 mol%), 0.533g:0.04g:0.2g (30 mol%), 0.533g:0.04g:0.3g (40 mol%), 0.533g:0.04g:0.4g (50 mol%), and 0.533g:0.04g:0.5g (60 mol%). The resulting bPEI:LiOH:PSSA (PLP) ternary mixtures were stirred at 60 °C for 15 h before the film coating processes. The GSP anode pastes for supercapacitors were prepared by mixing graphite (9.0 g), Super P-Li (0.3 g), and PVdF (0.7 g) in NMP (10 mg/ml).

Fabrication of supercapacitors: To fabricate asymmetric supercapacitors, indium tin oxide (ITO)-coated glass substrates were subjected to a patterning process to form ITO electrodes with dimensions of 12 mm × 8 mm (sheet resistance = 10 Ω/cm^2). The patterned ITO-glass substrates were thoroughly cleaned with acetone and isopropyl alcohol, dried under argon flow, and subsequently treated using a UV-ozone (UVO) cleaner (AC-6, AHTECH LTS Co., Ltd.) at a UVO intensity of 50 mW/cm^2 for 20 min. The GSP layers (~150 µm thick) were coated on the UVO-treated ITO-glass substrates and dried in a muffle furnace at 100 °C for 15 h. Next, the PLP electrolyte solutions were drop-cast on the GSP layers and soft-baked at 60 °C for 15 min, which led to the PLP SSE films. The same PLP SSE films were directly coated on the UVO-treated ITO-glass substrates (without GSP) (note that 150 μm-thick polyimide (PI) film spacers were mounted on the edge of the substrates for securing the thickness of the PLP films). Finally, the PLP SSE film-coated ITO-glass substrates were stacked on the PLP SSE film-coated GSP/ITO-glass substrates, which led to asymmetric supercapacitors with the configuration of glass/ITO/GSP/PLP/ITO/glass.

Measurements and analysis: The thickness of films was measured using a surface profilometer (Dektak XT, Bruker) and further confirmed from cross-sectional images using a field-emission scanning electron microscope (FE-SEM, S-4800, Hitachi). The surface morphology of film samples was analyzed using the same FE-SEM system. The core-level atom environment in the film samples was characterized by X-ray photoelectron spectroscopy (XPS, ESCALAB 250Xi, Thermo Scientific). A potentiostat (VersaSTAT4,

AMETEK) was employed for a series of electrochemical measurements, including cyclic voltammetry (CV), galvanostatic charge/discharge (GCD) test, and electrochemical impedance spectroscopy (EIS). The decay time constant (τ_D) was extracted by fitting the discharging curves with a single exponential decay equation, $V(t) = V_0 + \mathbf{exp}^{(-t \cdot \tau_D)}$, where V(t) and V_0 denote potentials at time = t and 0 s.

Table S1. Summary of previous reports on water-soluble polymer-based solid-state electrolytes for the application of supercapacitors.

No.	Water-Soluble Polymer	Li-Salt (content)	Electrolyte Compositions	Ion conductivity (mS/cm)	Tested Cycles	Hazardous Issues	Max. Potential (Applied Current)	Ref. (Year)
1	Poly(ethylene oxide)	-	Polymer/Nanosize Fillers	1.926	-	Halogen	2.0 V	SR-1 2015
2	Poly(vinyl alcohol)	LiTFSI (40 wt%)	Polymer/Salt/EMIT FSI	3.6	1000	Halogen	2.0 V (400 mA/g)	SR-2 2018
3	Poly(ethylene oxide)	$LiBF_4$ (5 mol%)	Polymer blend/Salt	0.2(RT)	1000	Halogen	0.5 V (1000 mA/g)	SR-3 2019
4	Poly(ethylene oxide)	$LiClO_4$ (40 wt%)	Polymer blend/Salt	0.734	100	Halogen	1.0 V (0.5 mA/cm^2)	SR-4 2019
5	Poly(vinyl alcohol) + chitosan	$LiClO_4$ (40 wt%)	Polymer/Salt	0.845	100	Halogen	0.9 V (0.5 mA/cm^2)	SR-5 2020
6	Corn starch	LiOAc (24 wt%)	Polymer/Salt/TiO_2	0.837	-	No	1.9 V (0. mA/cm^2)	SR-6 2021
7	Poly(ethylene oxide)	LiBr (4 mol%)	Polymer blend/Salt	0.375	500	Halogen	0.375	SR-7 2023
8	Branched-poly(ethylene imine) (bPEI)	LiOH (27 wt%)	Polymer/Salt	~1	700	No	0.9 V (0.4 mA/g)	SR-8 2023
9	Branched-poly(ethylene imine) (bPEI)	LiOH (27 wt%)	Polymer/Salt/ZnO	1.36	1000	No	2.0 V (0.4 mA/g)	SR-9 2024
10	Poly(ethylene oxide)	LLTO	Polymer blend/Salt	3.94	10000	Halogen	1.5 V (5000 mA/g)	SR-10 2024
11	Branched-poly(ethylene imine) (bPEI)	LiOH (55.5 wt%)	Polymer/Salt/PSSA	5.58	5000	No	2.24 V (0.2 mA/g)	This Work 2024

References for Table S1

[SR-1] N. K Singh, M. L. Verma, M. Minakshi, *Bull. Mater. Sci.* 38 (2015) 1577-1588.

[SR-2] J. Wang, Z. Zhao, S. Song, Q. Ma, R. Liu, *Polymers* 10 (2018) 1179.

[SR-3] B. Karaman, E. Çevik, A. Bozkurt, *Ionics* 25 (2019) 1773-1781.

[SR-4] S.B. Aziz, M.H. Hamsan, M. A. Brza, M.F.Z. Kadir, R.T. Abdulwahid, H. O. Ghareeb, H. J. Woo, *Results Phys.* 15 (2019) 102584.

[SR-5] M. Brza, S.B. Aziz, S. Raza Saeed, M.H. Hamsan, S.R. Majid, R.T. Abdulwahid, M.F.Z. Kadir, R.M. Abdullah, *Membranes (Basel)* 10 (2020) 381.

[SR-6] A. C. Ong, N. A. Shamsuri, S. N. Zaine, D. Panuh, M. F. Shukur, *Ionics* 27 (2021) 853-865.

[SR-7] S. Shenbagavalli, M. Muthuvinayagam, M. S. Revathy, *Ionics* 29 (2023) 211-231.

[SR-8] Y. Cho, S. Lee, H. Kim, Y. Kim, *J. Energy Storage* 57 (2023) 106010.

[SR-9] D. Murukadas, Y. Cho, W. Lee, S. Lee, H. Kim, Y. Kim, *Energy* 290 (2024) 129984.

[SR-10] R. Gurusamy, A. Lakshmanan, N. Srinivasan, S. Venkatachalam *J. Electroanal. Chem.* (2024) 118135.

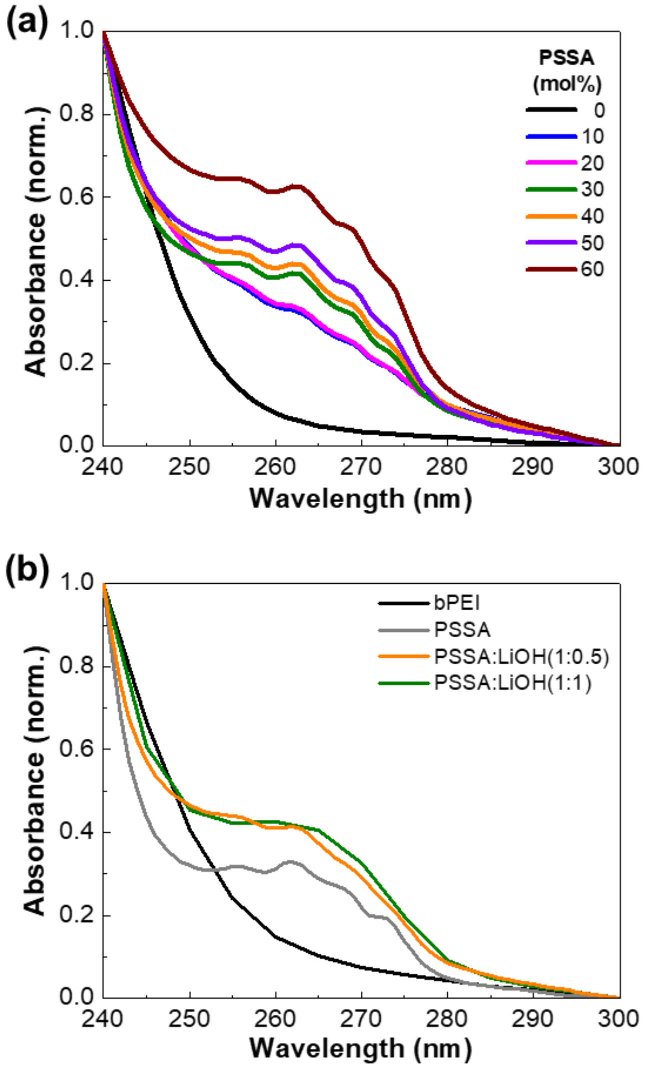

Figure S1. (a) Optical absorption spectra for the PLP SSE films (PSSA = 0, 10, 20, 30, 40, 50, 60 mol%). (b) Optical absorption spectra for the pristine bPEI film, the pristine PSSA film, and the PSSA:LiOH (1:0.5 and 1:1 by molar ratio) films.

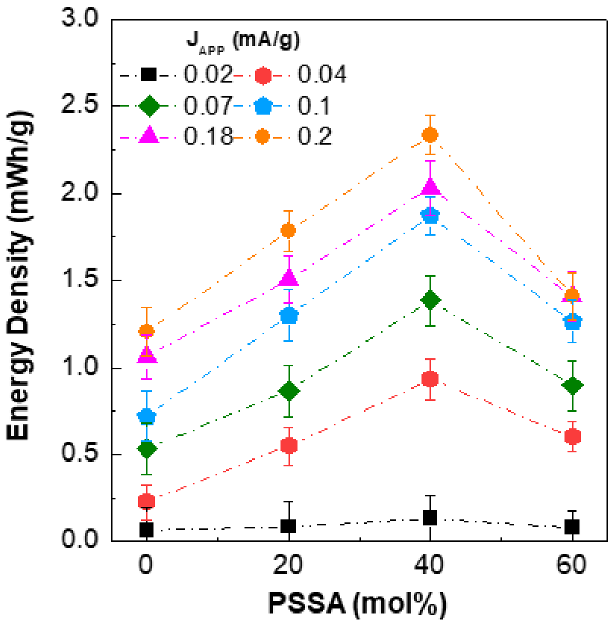

Figure S2. Energy density as a function of PSSA molar ratio for the supercapacitors with the PLP SSEs at various current densities applied consecutively (see the details in Figure 2a).

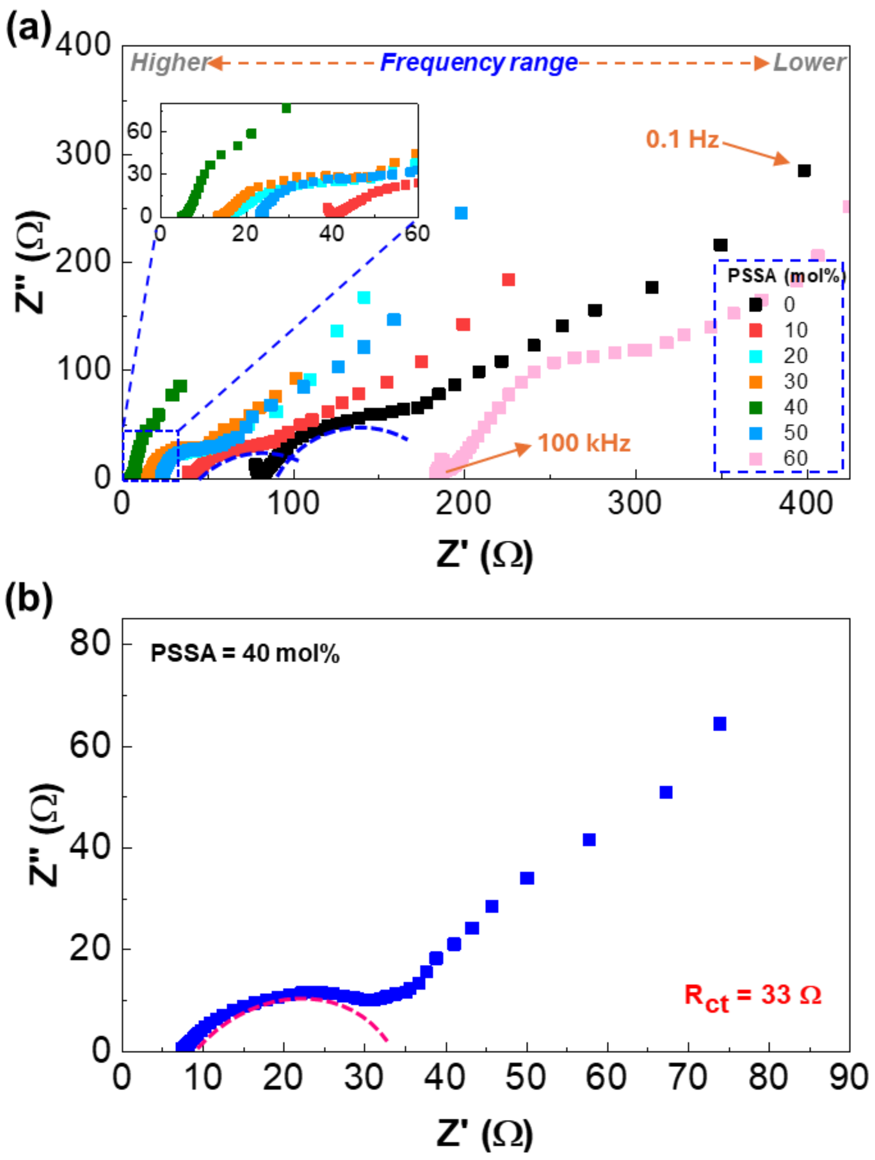

Figure S3. Nyquist plots of glass/ITO/GSP/PLP/ITO/glass devices: (a) according to the PSSA content (0-60 mol%) at 25 ºC (due to the Warburg impedance, the semi-circle parts are not so pronounced (see the enlarged parts for a better view of semi-circular areas), (b) PLP40-SC at 80 ºC. Note that the charge transfer resistance (R_{CT}) is typically obtained at the right end of semi-circles, which encounter the axis of the real part impedance (Z'). Accordingly, the ion conductivity (σ) can be calculated using $\sigma = t / (R_{CT} \times A)$, where t and A represent the thickness and surface area of the electrolyte films, respectively.

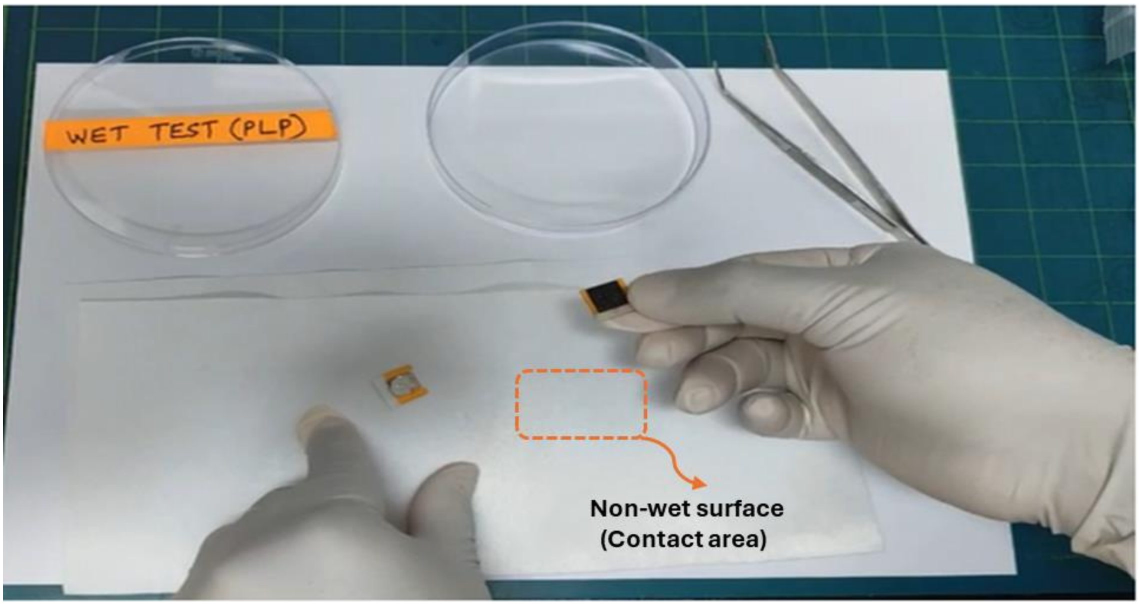

Figure S4. Wetting test of the PLP SSE films disassembled from devices by contacting with tissue papers. Note that no wetting spots were observed on the tissue paper after contact with both sides of the PLP SSE films (see the detail in the attached video clip: Fig_S4_video.mp4).

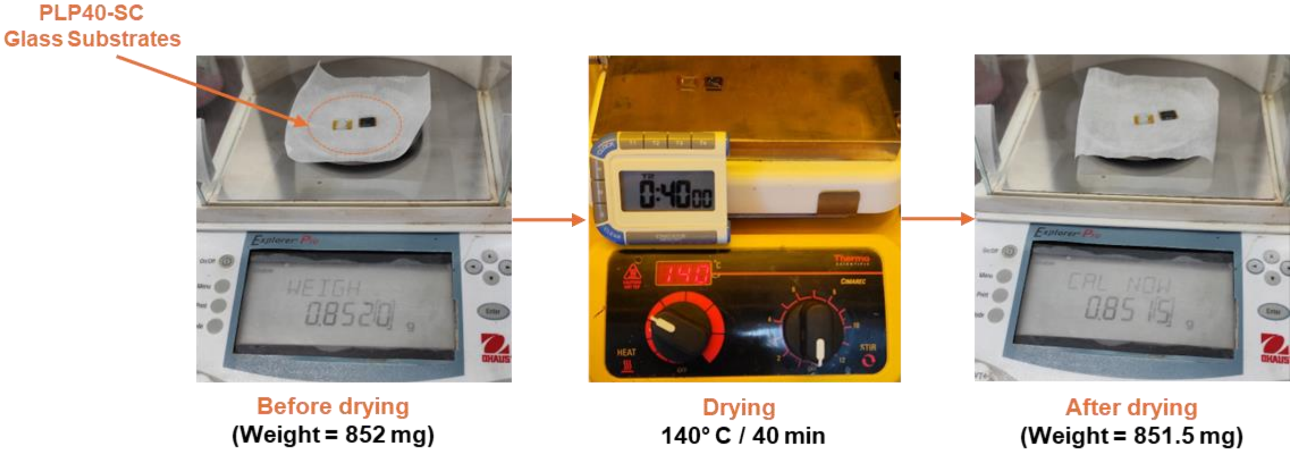

Figure S5. Brief gravimetric test for the weight change of the PLP film-coated substrate samples before and after drying at 140°C for 40 mins. The resulting weight change was around ca. 0.06% post-drying.

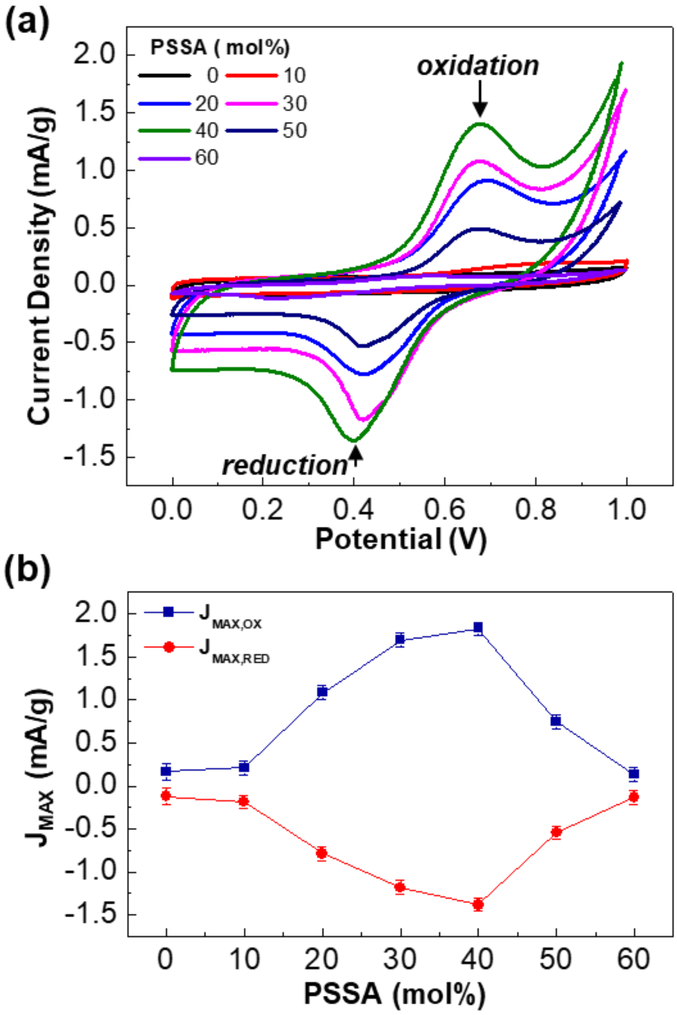

Figure S6. (a) Cyclic voltammetry (CV) curves for the supercapacitors with the PLP SSEs according to the PSSA molar ratio (sweep rate (SR) = 1.0 V/s). (b) Maximum current density (J_{MAX}) at the redox peaks in the CV curves in (a) as a function of PSSA molar ratio.

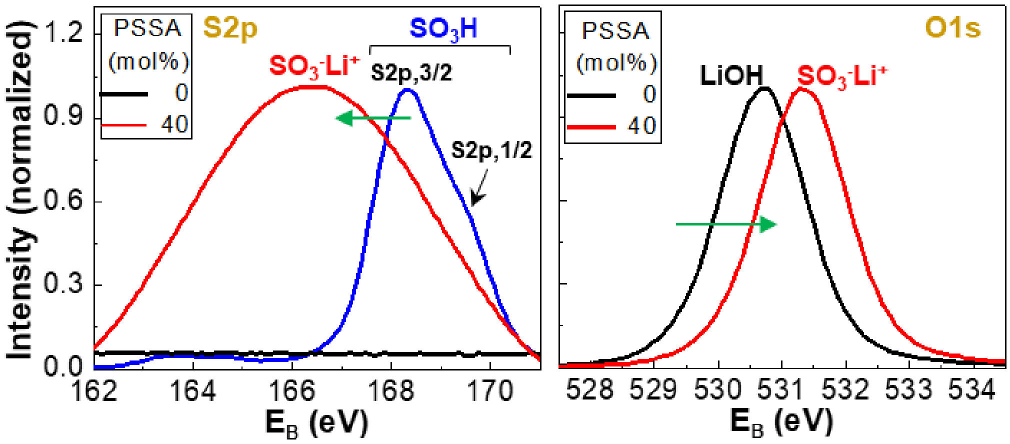

Figure S7. XPS spectra of the PLP SSE films (PSSA = 0 and 40 mol%): (left) S2p, (right) O1s. Note that both S2p and O1s peaks support the formation of lithium sulfonate (Li^+–SO_3^-) units and interactions in the PLP SSEs.

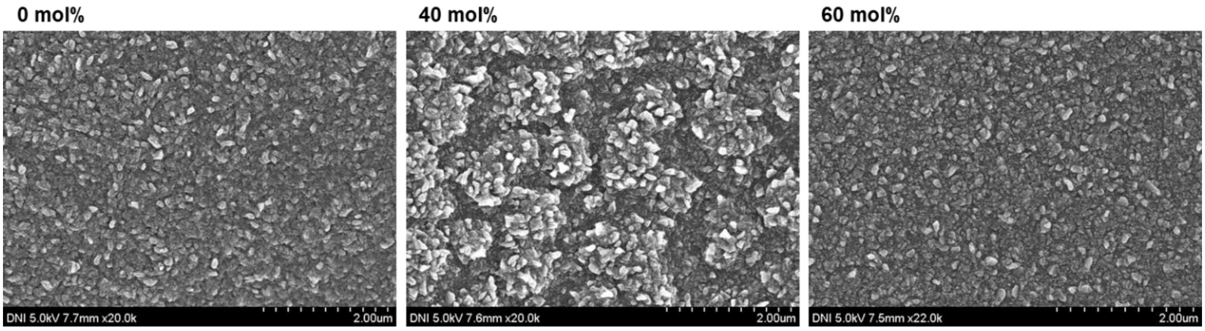

Figure S8. Magnified FE-SEM images for the surface of the PLP SSE films (PSSA = 0, 40, 60 mol%).

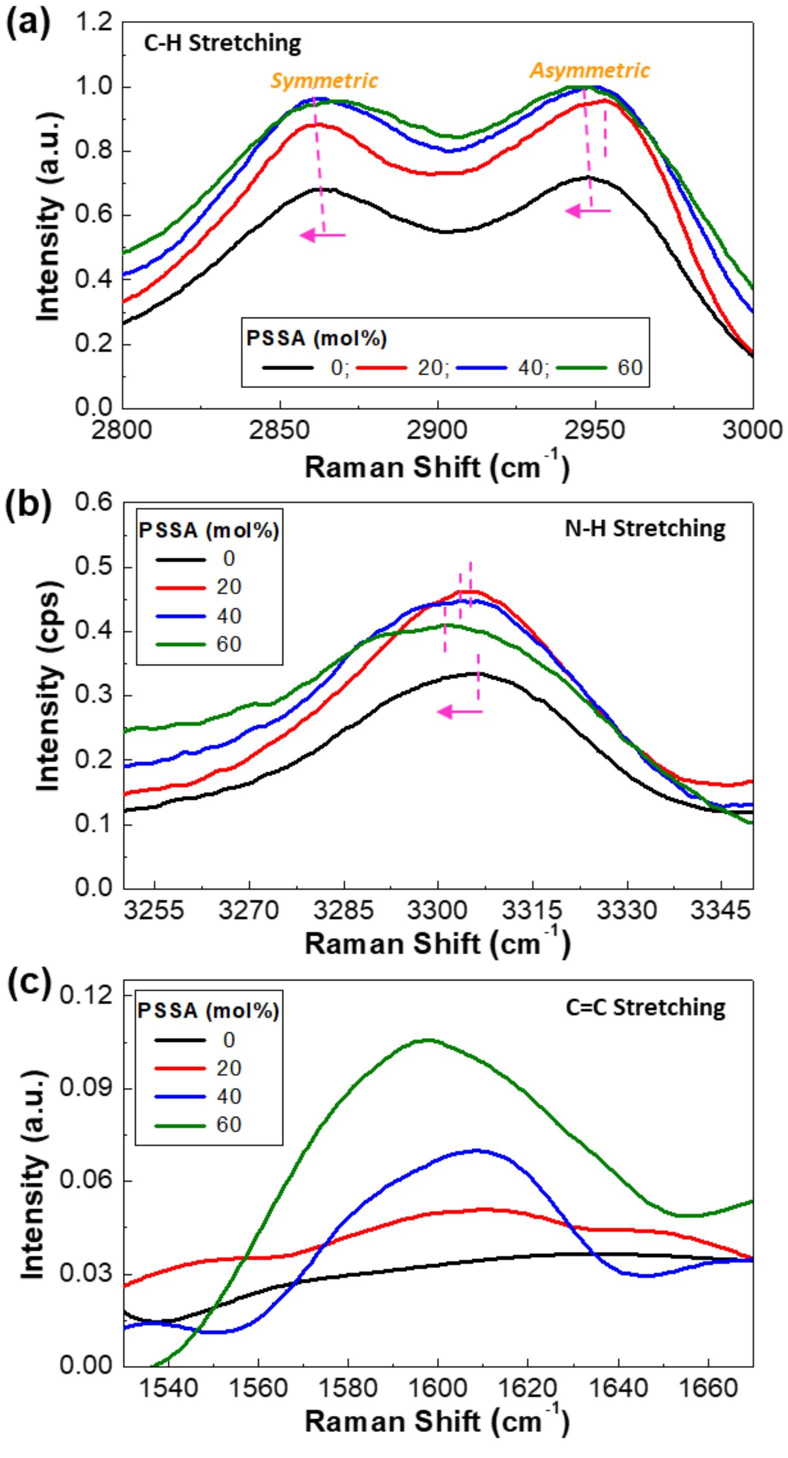

Figure S9. Raman spectra for the PLP SSE films according to the PSSA molar ratio (PSSA= 0, 20, 40 and 60 mol%): (a) C-H stretching, (b) N-H stretching, (c) C=C stretching.

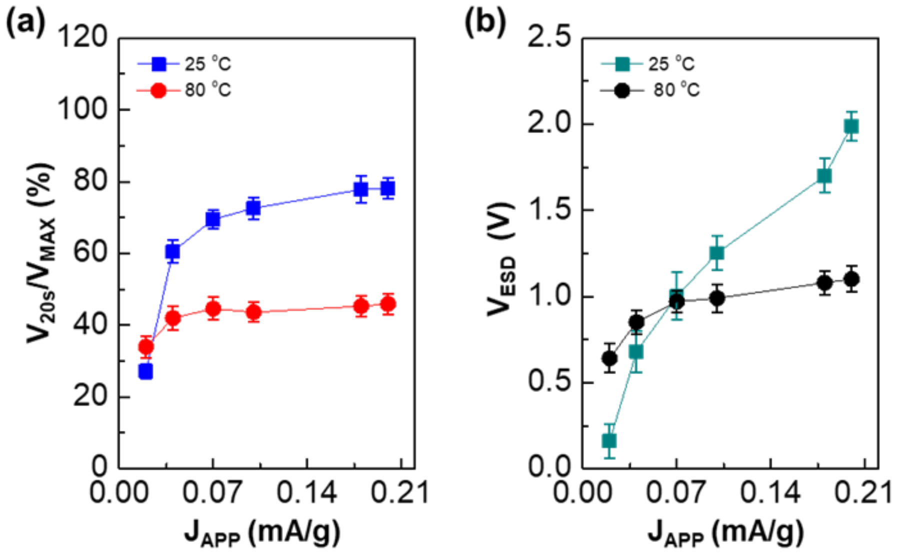

Figure S10. (a) Potential retention (V_{20s}/V_{MAX}) as a function of applied current density (J_{APP}) for the PLP40-SCs at 25 ºC and 80 ºC. (b) Electrostatic potential component (V_{ESD}) as a function of J_{APP} at 25 ºC and 80 ºC. Note that the electrostatic potential was rather reduced at 80 ºC when the PLP40-SCs operated at the higher J_{APP} despite the lessened potential retention.

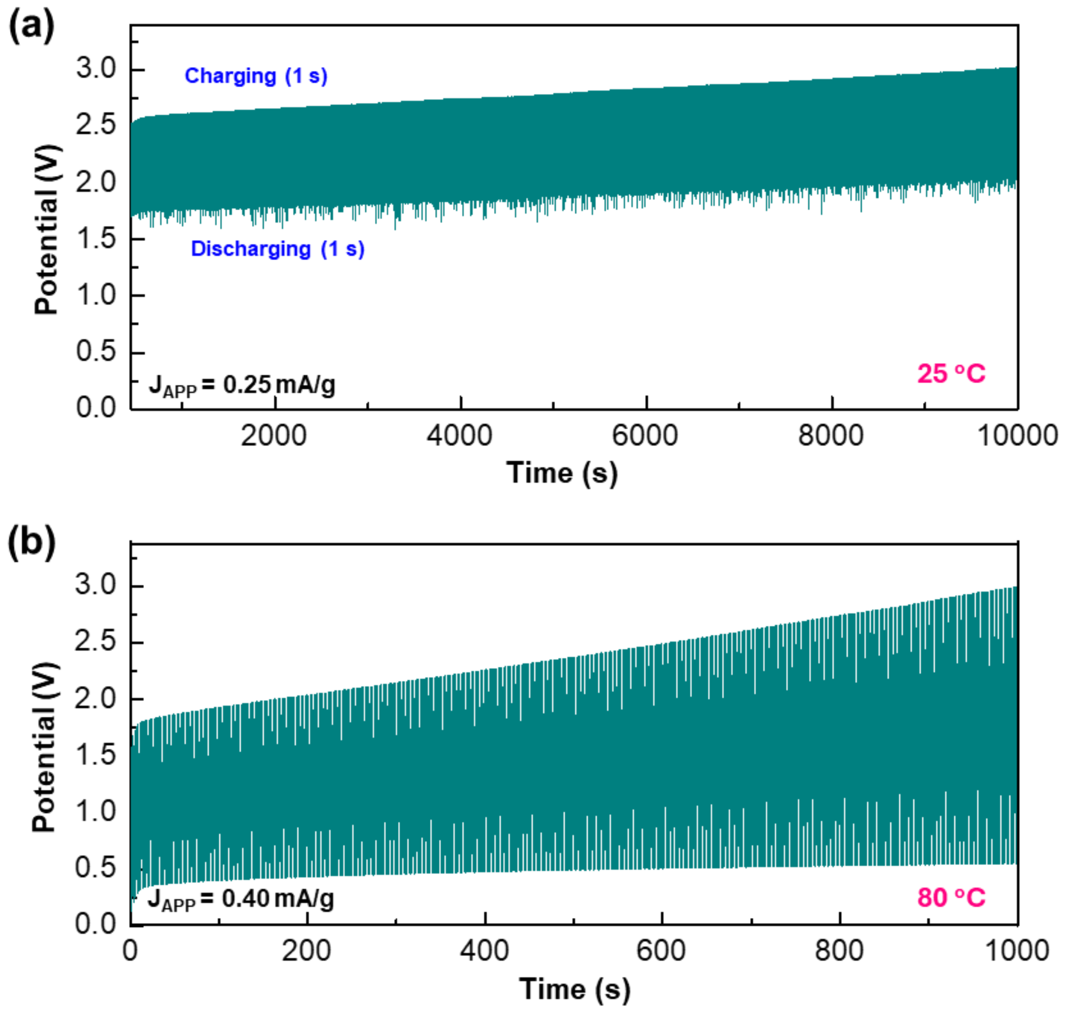

Figure S11. Change of potential as a function of operation time during long cycles of GCD test: (a) operation at 25 ºC (charging at J_{APP} = 0.25 mA/g for 1 s, discharging at J_{APP} = 0 mA/g for 1 s for 5000 cycles), (b) operation at 80 ºC (charging at J_{APP} = 0.40 mA/g for 1 s, discharging at J_{APP} = 0 mA/g for 1 s for 1000 cycles).

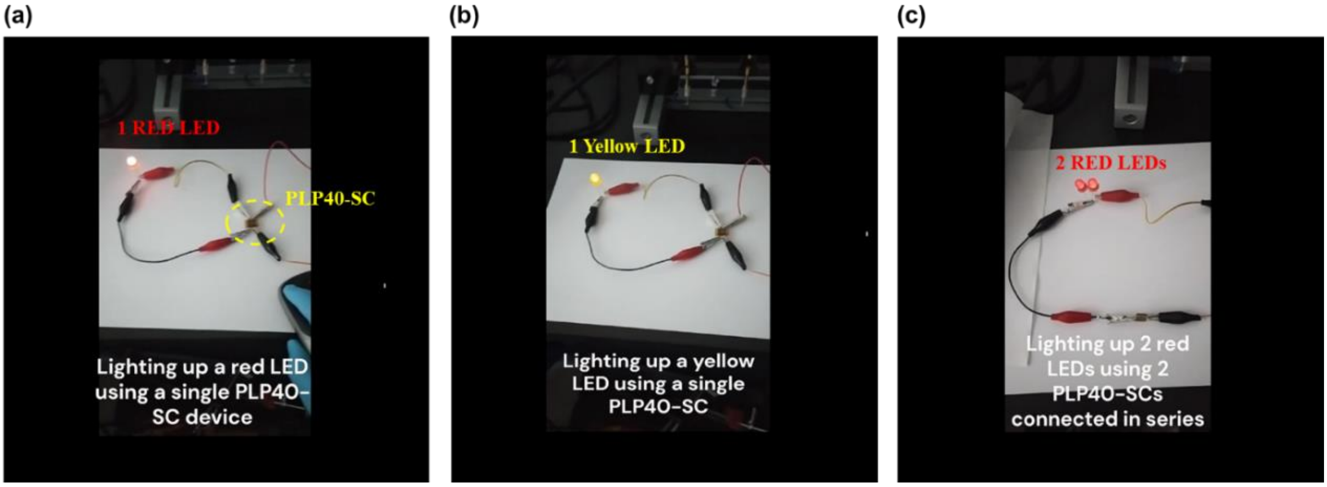

Figure S12. Operation of red and yellow LEDs using the present supercapacitors with the PLP SSEs (PSSA = 40 mol%) (PLP40-SCs): (a) Two red LEDs using two PLP40-SCs connected in series, (b) one red LED using one PLP40-SC, (c) one yellow LED using one PLP40-SC. Note that the charging (J_{APP} = 0.25 mA/g for 500 s) and discharging (J_{APP} = 0 mA/g) conditions were identically applied for all LED lighting tests (see video clips: Fig_S12a_video.mp4, Fig_S12b_video.mp4, and Fig_S12c_video.mp4).
